# Supplementary material for: Combinatorial ERK Inhibition Enhances MAPK Pathway Suppression in BRAF-Mutant Melanoma
Source: Int J Mol Sci. 2025 Oct 8;26(19):9794. doi: 10.3390/ijms26199794 (PMC12524744; doi:10.3390/ijms26199794)
Supplement: Supplementary file 1 [file ijms-26-09794-s001.zip › ijms-3616327-supplementary.pptx]

## Slide 1
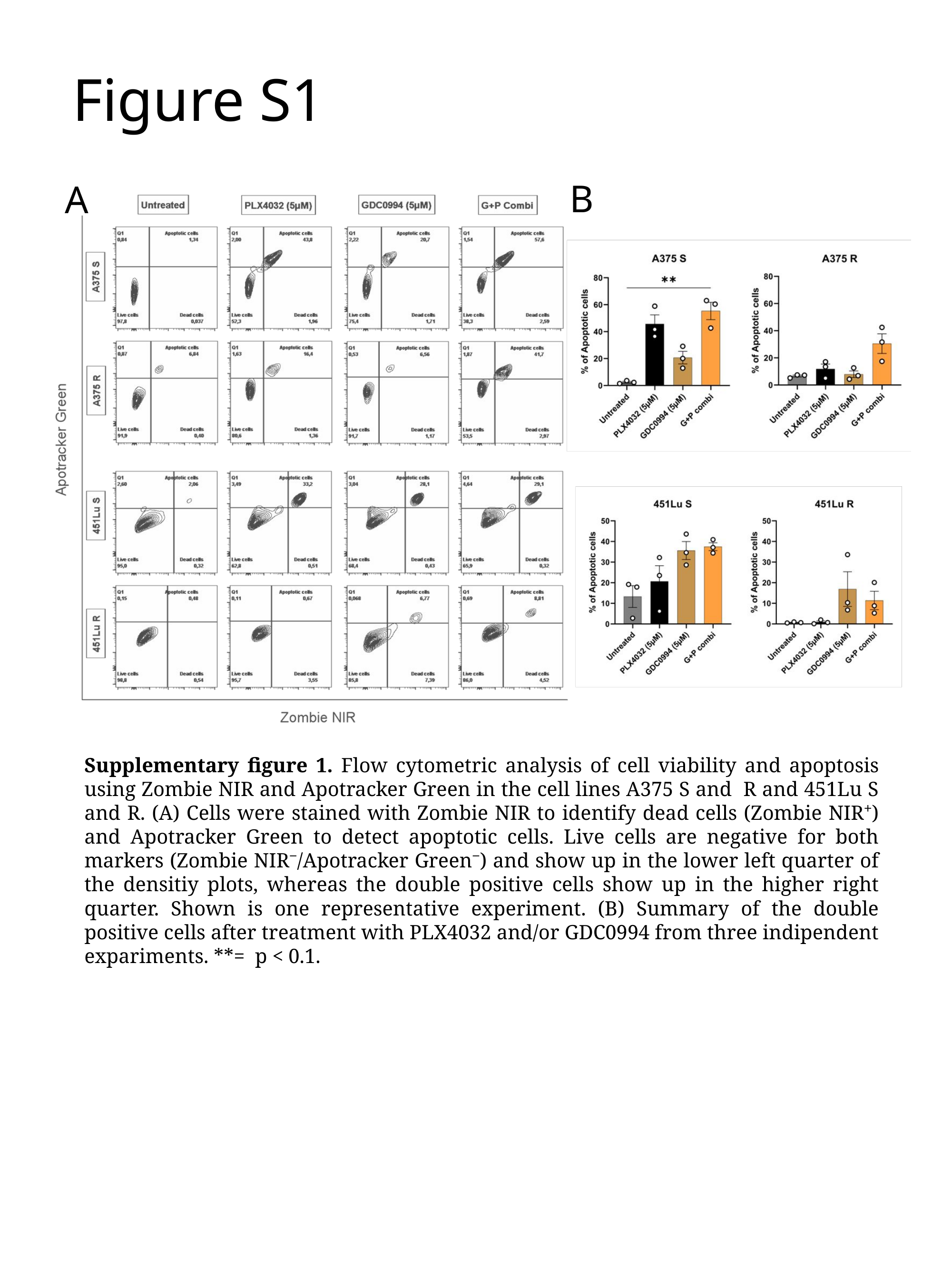

Figure S1
B
A
Supplementary figure 1. Flow cytometric analysis of cell viability and apoptosis using Zombie NIR and Apotracker Green in the cell lines A375 S and R and 451Lu S and R. (A) Cells were stained with Zombie NIR to identify dead cells (Zombie NIR⁺) and Apotracker Green to detect apoptotic cells. Live cells are negative for both markers (Zombie NIR⁻/Apotracker Green⁻) and show up in the lower left quarter of the densitiy plots, whereas the double positive cells show up in the higher right quarter. Shown is one representative experiment. (B) Summary of the double positive cells after treatment with PLX4032 and/or GDC0994 from three indipendent expariments. **= p < 0.1.

## Slide 2
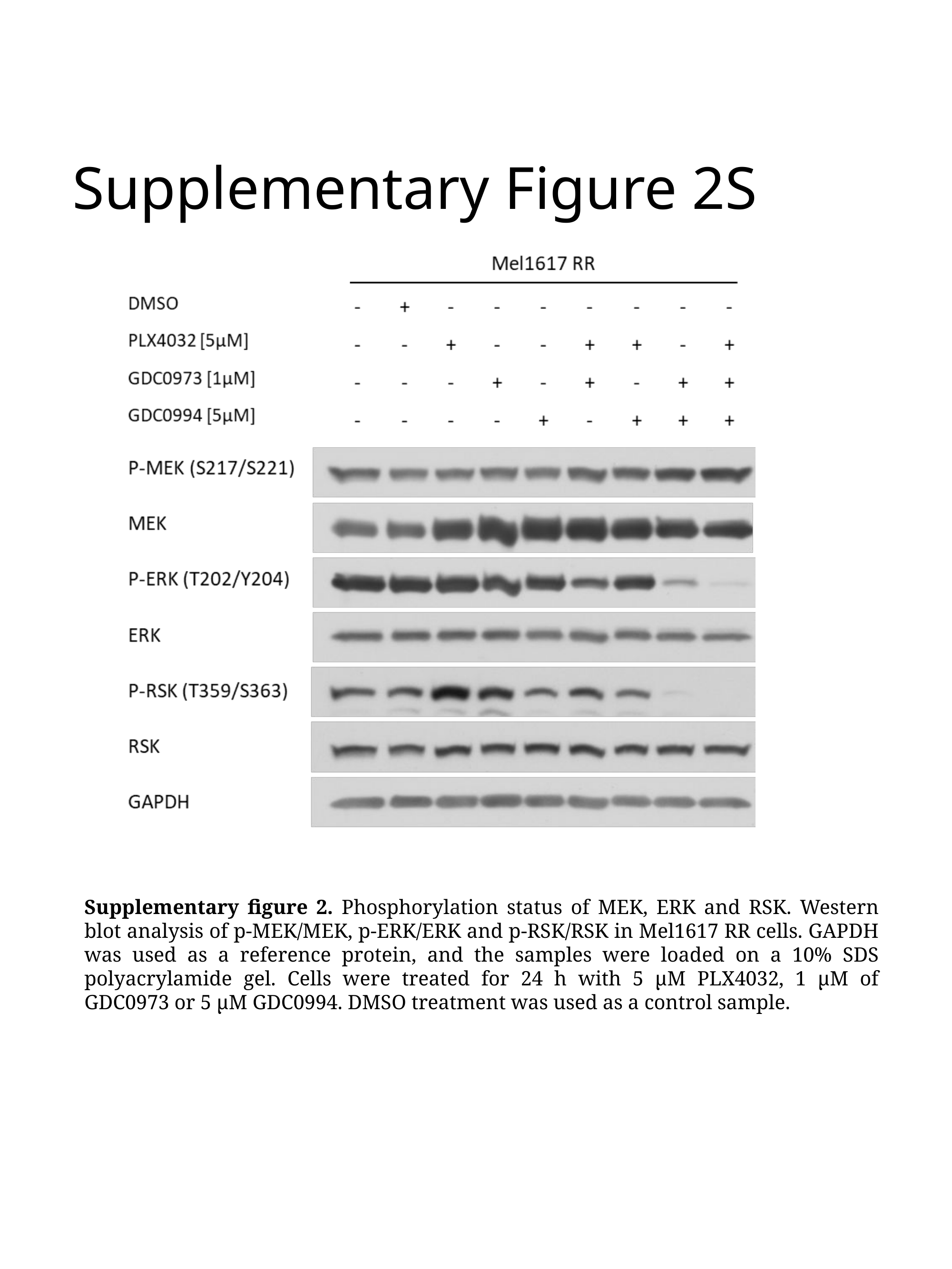

# Supplementary Figure 2S
Supplementary figure 2. Phosphorylation status of MEK, ERK and RSK. Western blot analysis of p-MEK/MEK, p-ERK/ERK and p-RSK/RSK in Mel1617 RR cells. GAPDH was used as a reference protein, and the samples were loaded on a 10% SDS polyacrylamide gel. Cells were treated for 24 h with 5 µM PLX4032, 1 µM of GDC0973 or 5 µM GDC0994. DMSO treatment was used as a control sample.
